# Severe personality disorder, treatment engagement and the Legal Aid, Sentencing and Punishment of Offenders Act 2012: what you need to know

**DOI:** 10.1080/14789949.2016.1155227

**Published:** 2016-03-31

**Authors:** Leon McRae

**Affiliations:** ^a^ Dickson Poon School of Law, King’s College London, UK

**Keywords:** Severe personality disorder, Treatment engagement, Indeterminate Sentence for Public Protection (IPP), Extended Determinate Sentence (EDS), Legal Aid, Sentencing and Punishment of Offenders (LASPO 2012), Offender Personality Disorder Pathway Implementation Plan

## Abstract

Empirical research has demonstrated a link between legal coercion and treatment engagement following conviction among those with severe personality disorder. Legal coercive pressures were often applied by the Indeterminate Sentence for Public Protection (IPP), until it was replaced by the Extended Determinate Sentence by the Legal Aid, Sentencing and Punishment of Offenders Act 2012. In this paper, it is proposed that use of the new determinate sentence will lessen motivation for treatment engagement. One effect of treatment refusal may be greater reliance by the Secretary of State for Justice on his jurisdiction to transfer prisoners due for release to secure hospital transfers under the Mental Health Act 1983. Not only will this risk posturing undermine the principal aim of the Offender Personality Disorder Implementation Pathway to improve treatment engagement among the target group, it will also have negative implications for medical practitioners working in secure forensic hospitals. To demonstrate what is at stake, the paper briefly recapitulates empirical findings familiar to readers of the journal, before drawing on original unpublished data.

## Introduction

In 2013, I reported on the findings of an Economic and Research Council (ESRC)-funded grant looking at the legal, therapeutic and interrelational implications of transferring those with antisocial personality disorder (ASPD) from prisons to an experienced secure personality disorder ward within the medium secure estate (McRae, 2013). The data were collected during the IPP-era, and shed light on the under-researched impact of indeterminate sentences on treatment engagement among offenders with ASPD. It was overwhelmingly found that offenders were engaging with treatment as part of an overarching strategy aimed at expediting parole (either during or following the expiry of their tariff period). For some, that strategy extended to seeking, and gaining entry, to medium secure units in order to overcome the problem of scarce prison resources connected to the Indeterminate Sentence for Public Protection (IPP). The long-term desire of this group was to convince the Parole Board that they had mitigated their risk through specialised treatment. Consider, for example, the following accounts:

[In hospital], you get on courses, boom, straight away. But in prison, there’s a big waiting list, you can wait years and years to get on courses … To be honest, I thought I’ll just give it a shot, and see what’s what. (Mr B) (ibid., p. 56)

I was kind of excited [by my pre-admission assessment], but I weren’t [*sic*] really as honest, well, I think I was perhaps too honest in the assessment … I probably exaggerated some of my answers … I knew what kind of stuff [the practitioners] were looking for. (Mr D) (McRae, 2015, p. 331)

My recommendation for sentencing was DSPD … I was waiting for a referral from [a DSPD prison unit] … They told me to expect to do 13 years, 13, 14 years. My tariff’s 3 years … I was told if I don’t do a DSPD, it’s highly unlikely I’m being paroled. So, that’s why I’ve come to this [hospital]. Hopefully, I can do enough [treatment] here to cancel out the DSPD. And I think I have. (Mr C) (ibid.)

While around 3633 offenders continue to be incapacitated in prisons by the IPP (as of 30 October 2014: Ministry of Justice [MJ], 2015), the sentence has not been passed for over three years. In its place, the Legal Aid, Sentencing and Punishment of Offenders Act 2012 (LASPO 2012) introduced a new Extended Determinate Sentence (EDS) for those ‘who would have previously been eligible for either an IPP or an Extended sentence for Public Protection (EPP) [for under 21s]’ (MJ, 2011a, para 25). The most important change introduced by the EDS is that offenders receive a prison release date. I argue in this paper that one unanticipated, and undiscussed, side effect of this change will be that it demotivates offenders from seeking out, or tolerating, treatment.

In making this argument, I do not assert that the IPP was reserved for those most in need of treatment, or that those with ASPD were the intended beneficiaries of the sentence; rather, I recapitulate empirical evidence demonstrating that the threat of coercion faced by those under the IPP – many of whom will meet a diagnosis for ASPD (Department of Health and Ministry of Justice [DH and MJ], 2011, p. 9) – appears to have encouraged them to seek out and tolerate treatment. Nevertheless, I intend no bold claims either about the suitability of sentencing law to induce treatment engagement among offenders or that its primary purpose is to affect it. As Lord Phillips of Worth Matravers CJ once put it: ‘The primary object of the IPP sentence [was] to protect the public, not to rehabilitate the offender’ (*Walker and James v Secretary of State* [2008] EWCA Civ 30, para 69). However, in this judgment, he was bound to concede: ‘The evidence is that prisoners serving [the] IPP have no realistic prospect of being released at the direction of the Parole Board unless they have attended relevant offending behaviour programmes …’ (ibid., para 15). My position is therefore, necessarily, pragmatic: an inherent benefit of the coercively framed IPP was that it could encourage treatment engagement.

Outside the sentencing context, concern about poor treatment motivation and offender apathy towards treatment has recently been expressed in the Offender Personality Disorder Pathway Implementation Plan (OPDPP: DH and MJ, 2011). In brief, the OPDPP outlines plans to reinvest funds previously pumped into the dangerous and severe personality disorder (DSPD) pilot into the high-secure prison estate for those with severe personality disorder (SPD). The aim of the OPDPP is ostensibly simple: to provide offenders with specialist treatment ‘to help them lead less destructive lives’ (ibid., p. 6). With this goal in mind, interested medical have taken up the challenge of working with this group by enrolling on various educational programmes, from diploma to PhD level, as part of the Knowledge and Understanding Framework (KUF: http://www.personalitydisorderkuf.org.uk/index.html) run in conjunction with the Personality Disorder Institute in Nottingham. This coalescing of talented and dedicated medical practitioners for a population once recognised as neglected (National Institute of Mental Health in England [NIMH(E)], 2003) is a notable success of the initiative. A possible benefit of knowledge exchange is that issues of staff burnout, low morale, stress and managerialism may be better managed than hitherto:

Someone was brought crying their eyes out because they were being moved to [the personality disorder ward for patients from Rampton]. Their interest is mental health … and they’ve made people aware, rather than personality disorder. (Nurse)

In this paper, however, I argue that the laudable aim of improving treatment engagement among SPD-offenders and improving workforce capacities will be undermined by the introduction of the EDS. An important, and potentially disastrous, consequence of poor treatment engagement caused by the EDS may be increased reliance by the Secretary of State for Justice on his jurisdiction to transfer sentenced prisoners who have reached their earliest release date to hospital for ‘treatment’ under section 47 of the Mental Health Act 1983.

Elsewhere, I have attempted to answer this possible side effect by proposing further sentencing reform and enthusing medical professionals to inveigh against late transfers (McRae, 2015). Here, my aim is to elucidate those aspects of the EDS and OPDPP that indicate the need for these implicit, co-optive responses. The problem, I argue, is that, unlike the former Labour government who considered, if in brief, the potential impact of the IPP on treatment take-up in DSPD units (Ministry of Justice, 2005), the coalition government failed to acknowledge the empirically proven link between legal coercion (through indeterminate sentencing) and treatment motivation among SPD-offenders (ibid.).

## LASPO 2012: Disjuncture between the EDS and offender responsibilisation

In 2012, the European Court of Human Rights heard the case of *James, Wells & Lee v The United Kingdom*, App. Nos. 25119/09, 57715/09, and 57877/09 [2012] ECHR 1706. The applicants alleged, *inter alia*, that the failure to provide treatment courses required by their IPP sentence plan was unlawful under Article 5(1)(a) of the European Convention on Human Rights. The ECtHR concurred, ruling that, while the fact that rehabilitation was not ‘an express [statutory] objective’ of the IPP, the sentence had to provide ‘a real opportunity for rehabilitation is a necessary element of any part of the detention which is to be justified solely by reference to public protection’ (para 209). In the aftermath of the judgment (though not explicitly acknowledged as a catalyst by government), the LASPO 2012 was enacted to amend the Criminal Justice Act 2003. One effect was the abolition of the IPP on 3 December 2012 (section 123 of the LASPO 2012), which was replaced by the EDS.

In contrast to indeterminate sentences, offenders receiving the EDS are released from custody at the two-thirds point in their sentence. The one caveat is that offenders serving a custodial term of 10 years of more – or those who have not previously been sentenced to a term of 10 years or more, in which case a discretionary life sentence can be passed – will be released on reaching their full term, unless the Parole Board authorises release at the two-thirds point. This means that, although the sentences under the amended Criminal Justice Act 2003 are generally longer compared to determinate sentences passed during the IPP-era (MJ, 2014b, p. 23), EDS-offenders are guaranteed a release date. If released prior to the completion of the sentence, EDS-offenders placed on licence: a maximum of 5 years for a violent offence and 8 years for a sexual offence (section 226A (8)).

Using prison population data as a best estimate, the number of offenders receiving the EDS stands at 1,359 offenders between 3 December 2012 and 31 December 2014 (MJ, 2014a, p. 23), with a disproportionate increase of around 460 since March 2014 (MJ, 2014b, p. 15). The average custodial sentence length is of 8.1 years (MJ, 2014a, p. 23). So, what does this mean for the ASPD population?

During the IPP-era, the Sainsbury Centre for Mental Health (SCMH) (2008) found that 66% of offenders serving the sentence required clinical assessment for personality disorder. This indicates that a similarly high proportion of EDS-offenders meet diagnostic criteria for (anti-) social personality disorder, with all the therapeutic uncertainties and interrelational tensions that attend. The coalition government was, nevertheless, silent on the relationship between ASPD and the EDS. As part of the bland strategy aimed at ‘ensuring that justice is clearly seen to be done’ (MJ, 2011a, para 33), it was asserted that ‘All offenders under [this] sentence will be required to follow a sentence plan including undertaking a range of targeted rehabilitative interventions, aimed at reducing their risk’ (ibid., para 27). The closest we come to an appreciation of the complexities involved in rehabilitating this group is their observation that the success of ‘rehabilitative interventions’ is predicated on ‘offenders taking responsibility and action to reform’ (ibid., para 35).

Despite the absence of careful consideration of the implications of having ASPD in this new sentencing context, we have been given assurances by the coalition government that ‘all methods of effective rehabilitation [will be] considered and used …’ (ibid., para 35). But nowhere in their account of offender responsibilisation and rehabilitation is a frank discussion of the relationship between treatment take-up and sentencing context. Below, I argue that two linked ill-effects may result from the government’s deprivileging of sentencing context from treatment provision and engagement. First, plans contained in the OPDPP to improve treatment engagement among offenders in the high-secure prison estate will be undermined. Second, the public protection response may to increase late hospital transfers of determinate sentence offenders under section 47 of the Mental Health Act 1983.

## Rereading the OPPDP through the lens of the EDS

The OPPDP justifies plans to reinvest funding from DSPD units to the high-secure prison estate on the basis that more treatment places will be available for SPD-offenders: up from around 350 under DSPD hospitals and prisons to 570 in Category A prisons. Further, 820 aftercare places in Psychologically Informed Planned Environments (PIPES) will be available for those who have completed a period of treatment (including those within the ‘pathway’), so that changes in behaviour can be overseen in the community.

The underlying policy ambition is to improve self-responsibility among offenders with SPD and, ultimately, public protection by identifying those with ‘a minimum of three years still to serve’ (ibid., p. 18). Long term, provided spending plans remained unchanged under the conservative government, ‘pre-pathway’ facilities will eventually be implemented to improve treatment motivation among newly sentenced offenders through collaboration between professionals on matters of ‘case formulation and sentence planning’ (ibid., p. 9).

While continued efforts to invest in a marginalised population will be welcomed by those working in the field, the details of how existing SPD-offenders will be identified are extremely sketchy. Both close supervision centres and those with many prison adjudications are identified as sources of complex cases needing treatment. However, the OPDPP is clear that ‘where prisoners meet the criteria of the Mental Health Act they will be detained in hospital’ (DH and MJ, 2011, p. 9). Therefore, if close supervision centres are, even in part, relied upon, the ‘high levels of self-harming behaviours due to [the population’s] clinical needs’ presented there (Hodson, quoted in Allison, 2011) would presumably preclude admission to ‘the pathway’. By comparison, how do you discriminate between large numbers of prisoners with numerous adjudications? Are there sufficient resources to screen all those causing interrelational problems in prison? Surely not. If, instead, resources are poured into screening at the sentencing stage, there is the unreliability of pre-sentence reports to contend with:

There are many [cases] where those [psychiatric] recommendations are not made [at sentencing] and you could easily say could be. (Psychologist) (McRae, 2015, p. 333).

At the outset, then, it is clear that many will not be identified for ‘the pathway’ who might most need treatment. Between those who are identified and those who are not, emphasis must be placed on offenders seeking treatment. In the context of hospital admissions during the IPP-era, this was accepted practice (at least outside the high-secure estate). Mr C, for example, states:

[The prison psychiatrist] was telling me I wasn’t suitable for [this hospital] … Then, one day, I thought fuck it … I got in touch with my solicitor and they commissioned for reports to be done, and he put the reports forward [to this hospital], and that was good enough. [The staff] came to see me, assess me … My behaviour controls my release date (ibid., p. 322)

Unfortunately, the OPDPP has unwittingly delinked the importance of sentencing context from the achievement of motivating offenders. Early parole is clearly an important goal of anyone detained; legal coercion, whether via the IPP or otherwise, acts as a potent source of external motivation for SPD-offenders. Even now, personal correspondence with forensic psychiatrists working in the field indicates that personality disorder wards (at least of medium security) are accommodating indeterminate sentence prisoners. It seems optimistic to assume that these wards will come to be filled by determinate sentence prisoners equally eager to engage in a potentially therapeutic process.

This limitation of the EDS is most acute for those whose sentence is less than 10 years: unless the offender is genuinely motivated to change his or her self-understanding (and there is, as yet, little reason to assume this is the majority), the expectation of prison release at the two-thirds point of his or her sentence will weaken desire for treatment. By comparison, if the offender has received a sentence of over 10 years, he may decide to seek treatment when the completion of a few years of specialist treatment coincides with the two-thirds point of his sentence. Expecting him to self-identify early in his sentence for high-secure prison services is unrealistic. We would have to assume that SPD-offenders understand the extent of the current risk of incapacitation, absent statistical or anecdotal evidence in research. An appreciation of the risk may only arise once the possibility of increased hospital transfers presented in this paper has been realised. Their engagement will be reactive rather than pre-emptive. Furthermore, submission to treatment would also assume the offender perceives a clear link between receiving treatment and expedited parole. Some may, indeed, have experience of being released post-treatment following the expiration of their tariff period under the IPP; others in this group will have suffered incapacitation, perhaps in hospital, and perceive a fictive relationship between treatment and release. For instance, Mr E reflects:

I still have me [*sic*] ups and downs, we all have our ups and downs. It’s difficult anyway being an IPP prisoner or a lifer, because all of us here are lifers or IPPs. When you’re on that type of prisoner, it’s a killer because alls you’ve got is your hope and motivation, you’ve got no date to look forward to, nothing.

Under the EDS, if offenders continued to be enticed by medium secure *hospitals*, it is likely to be for reasons unrelated to a true desire to change criminogenic outlook. Consider, for instance:

(1) The interrelational tensions in prison, particular with staff, may encourage the belief that the hospital will be a more humane than prison:

Mr M states: I couldn’t ask the prison officer for help. You couldn’t be seen as weak. In that environment, it’s them and us. You don’t play chess, you don’t play pool, you don’t play fucking scrabble with the prison officers. The moment you do, you’re labelled a wrong’un, a grass, and then you’re open to target, everyone wants ya. (McRae, 2013, p. 57.)

(2) They may wish to achieve (escorted or unescorted) leave under the MHA (section 17 of the MHA);
(3) They may (arguably, falsely) assume that release from hospital offers a better opportunity for aftercare:

Mr L states: If you get released from [hospital], you get a massive release package. You go into residential care, where there is someone at hand if you’re having problems all the time. You get a [Community Psychiatric Nurse]. You’re known to social services. You get put on invalidity care benefits. You get given so much. You get more chance of being given a house, accommodation. (ibid., p. 334)

In view of the ostensible aim of the OPDPP of encouraging changes in self-understanding in SPD-offenders, it remains an important task of empirical and clinical investigation to uncover the extent to which, if any, motivation to enter treatment due to sentencing context might transmute into a true desire to change once the offender is exposed to psychiatric norms through treatment. A key challenge for treatment providers continues to be identifying whether treatment has actually been successful in reducing criminogenic risk. With the potential for offenders to enter and engage with treatment strategically (for discussion, see McRae, 2013, 2015), this is no easy task. Particularly, at the early stages of admission, an offender who presents as motivated by the process of change during pre-admission assessment may subsequently experience difficulty acculturating to the hospital environment. For example, one nurse comments:

[They’ll] present as very motivated in the [pre-admission assessment], and you’ll feel quite positive about them. But when they get here, they just can’t handle it. They’ll fail in different ways. Some will decide they want to go back to prison themselves, because it’s not for them at that time, or they’ll go down the route of being violent to get back to prison. (McRae, 2015, p. 334)

In the past, (remorseful) offenders may have returned to hospital after a ‘period of reflection’ for further treatment (ibid., p. 55). Readmission in these circumstances inevitably encouraged belief that changes in self-understanding were possible, or indeed manifest. A psychiatrist posits: ‘[P]eople who aren’t particularly motivated at the beginning have a sense, you know, I get what this is about and I want to do it properly’ (ibid., p. 58). While I believe an effective treatment dose may be achieved over time, even in the continued presence of ulterior motives for engagement (McRae, 2013, pp. 60–62), this possibility relies on SPD-offenders identifying treatment engagement with their own best interests. Or, as has elsewhere been suggested, choice (whether or not false consciousness induced by psychiatric norms) rather than coercion – which has the potential to 'mobilize his or her energies and resources in order to accomplish the treatment goal' (Winick, 1994, p. 105). In addition to perceiving a clear pathway into the community, the offender’s preference of treatment location clearly prefigures the choice to engage, and therefore the potential for changes in self-understanding that might result from treatment.

While a thorough exposition of the ability of treatment norms to effect behaviour change would clearly take us too far afield from the issue of sentencing, it is noteworthy that the OPDPP appears to be cynical of second attempts at treatment outside the high-secure prison estate. Offenders who return to prison in the manner described above will unwittingly fall within the category of offender identified by the OPDPP as ‘unlikely to make progress in other interventions’ (DH and MJ, 2011, p. 18). If this becomes the route through which some, if not many, offenders are identified for ‘the pathway’, those serving a sentence of 10 years who are nearing the two-thirds point in their sentence *may* equate engagement with an opportunity for parole before completion of the full term. In sharp contrast, those serving sentences less than 10 years who are close to their date of release will eschew treatment.

Based on past experiences, I argue that this scenario may encourage increased use of section 47 of the MHA by the Secretary of State for Justice, who will be concerned about public protection issues if treatment naïve offenders are released from custody. I expand on this hypothesis below.

## Late hospital transfers: an unfortunate remedy for treatment naivety

The practice of transferring determinate sentence offenders to hospital at their release date is well documented. As early as 1989, the Mental Health Act Commission (MHAC, whose responsibilities have now passed to the Care Quality Commission (CQC)) drew attention to the practice in its Third Biennial Report 1987–1989 (see also Grounds, 1990). Some years hence, the interrelational and therapeutic impact of doing so was outlined (MHAC, 2008, para 7.19):

At Broadmoor hospital in June 2007, we noted a number of [late transfer] patients who were refusing to engage in therapy and otherwise proving to be a severe management problem due to their resentment at such late transfers. One patient told us that he had been assessed several times in prison and told that he did not have a mental disorder, before his sudden transfer towards the end of his sentence.

While the impact of late transfers will not always be therapeutically disastrous, the desire among medical practitioners to cultivate an environment conducive to therapeutic gain is always jeopardised when it occurs. In an effort to minimise disruption, the responsible clinician, in conjunction with her treatment team, is at liberty to consider releasing offenders who have reached his or her ‘prison’ release date. With EDS-offenders, however, the availability of licence periods for those released may delimit medical discretion. Morris, Gibbon, and Duggan (2007) provide an example of this phenomenon in the case of a late transfer to a medium secure unit of a determinate sentence prisoner during the IPP-era:

Although [Mr A] appeared to be reassured by [our plans to discharge him with a care plan in the event of disengagement], it had the opposite effect on those who wished him to be detained. Consequently, a condition was written into his probation license so that if he was discharged as a result of his disengagement, this would be interpreted as a breach of his license so that he would immediately be returned to prison. He was on license for a further six months.

In this situation, the only option left for the offender is to test the legality of his continued detention by applying to a first-tier tribunal (Mental Health). An up-to-date medical report favouring discharge, presumably with a suitable aftercare package (as in the case of Mr A, above), will increase his chances of release. In other situations, the responsible clinician, while presumably concerned about the offender’s placement on the ward, may not support discharge out of fear of being held accountable if things go wrong in the community. While, by comparison, the fact-finding process undertaken by the tribunal ensures limited therapeutic jurisprudence, it has been argued that a humane response by the tribunal to the deprivation of liberty is ‘for a clear determination to be made upon [what] formed the view that emergency intervention was necessary' (Freckelton, 2003, p.52). This could include making informal pronouncements about the unfavourable circumstances of the offender’s admission to the hospital ward. Empirical research already reveals concern among tribunal members about late transfers upon which pronouncements could be based:

What’s uncomfortable about the present system is that [the Secretary of State] can shift [the offender] into [this] system, which is effectively a life sentence … the Court has already sentenced [offender] properly for the offence so [the Secretary of State is] is re-sentencing [him] effectively. And I can’t see that that’s right. (MHRT12, Medical member) (Trebilcock & Weaver, 2009, pp. 74, 75).

This manoeuvre is particularly important given that late transfers appear unimpeachable. In the case of *R (on the application of SP) v Secretary of State for Justice* [2010] EWCA Civ 1590, the Court of Appeal considered whether medical reports, which had not considered the issue of whether treatment would have any benefit, were suitable for the purposes of authorising a late transfer. Lady Justice Arden replied that the Secretary of State is only concerned to see whether ‘the medical practitioners have given some reasons which they consider adequate’ (para 23) to support admission. This is unfortunate insofar as Lord Justice Waller in the earlier case of *R v Secretary of State for Justice, ex parte TF* [2008] EWCA Civ 1457 stated that late transfers should occur only in ‘very exceptional circumstances’ (para 31), and that there might be human rights arguments raised if transfers were effected simply because ‘a convicted person will be a danger to the public if released (as understandable as that concern must be)’ (para 18). However, this judgment was limited in scope by the removal of the MHA ‘treatability test’ in October 2008. Since then, ‘appropriate treatment’ (section 3(2) of the MHA) is that which has the ‘purpose’ of addressing the respective mental disorder or its symptoms (section 145(4)).

Nevertheless, more recently the Department of Health (DH) (2011) issued guidance that the Secretary of State for Justice will not consider transferring the prisoner unless:

• Admission to hospital is an urgent necessity;
• It is necessary for the prisoner’s own health and/or safety and
• The urgency of need is such that it is not safe to wait until the release date for admission to hospital (para 3.13).

However, there is an uncomfortable tautology to these ‘requirements’: the ‘urgent necessity’ to transfer, which cannot wait until the offender is released (and admission effected under civil section), is being defined by the offender’s criminal past, while that past implies fulfilment of the transfer conditions. The requirements also imply that late transfers take effect because medical practitioners are directing them. While admissions must be recommended by medical practitioners under section 12(2) of the MHA, the Ministry of Justice guidance is clear that the conditions that must be fulfilled are essentially those of the MHA:

• The prisoner is suffering from mental disorder;
• The mental disorder is of a nature or degree which makes it appropriate for the prisoner to be detained in hospital for medical treatment;
• Appropriate medical treatment is available; and,
• There is an urgent need for treatment for unsentenced prisoners.

Again, urgency of admission will be easily implied by the offender’s criminogenic character, which is adequately denoted by the nexus of diagnosis and public protection concerns. This indicates that the practice of late transfers will continue to be the prerogative of the Secretary of State, whatever the guidance issued by the Ministry of Justice suggests. Indeed, Bartlett and Sandland (2014) recently confirmed that late transfers continue to occur.

A potential rejoinder to this analysis is the claim that there are insufficient beds to cater for an increase in transfers to the secure estate (see e.g. Lord Bradley, 2009). Consider, in 2015, the CQC reported that the number of NHS beds decreased by 8% between 2010–2011 and 2014–2015. However, this shortfall probably results from contractual pressures to maintain occupancy at 90% or above (Centre for Mental Health [CMH] 2011). Even so, it has been reported that empty beds on wards remain possible in specialised personality disorder units:

The interesting thing is how few referrals we get, not how many … Most of the time, we haven’t got enough people to fill 12 beds in a population of 5 million or so. (Psychiatrist) (McRae, 2015, p. 332)

Even in areas, such as London, which experiences dynamic populations in specialist tertiary services, *fluctuating* occupancy is reported (HM Chief Inspector of Prisons for England & Wales, 2013). In such circumstances, SPD-offenders perceived by the Secretary of State to be risky will find little competition for beds from offenders (perhaps with other forms of mental disorder) still serving a custodial sentence. The result is that the interrelational and therapeutic concerns expressed in this paper would remain acute. While we await with interest preliminary reflections on the success of the OPDPP, the introduction of the LASPO 2012 suggests that it may become an impoverished successor to the problematic DSPD pilot.

## Conclusion

The introduction of the LASPO 2012 stands to challenge the belief that offenders with SPD can be inspired to engage in treatment to mitigate their risk, absent legal coercion. Empirical research clearly reveals a link between coercive sentencing and offenders’ willingness to engage in treatment. In the past, the coercive element was provided by the IPP; in future, the EDS is set to disincentivise the very group perceived by the government to the most needed treatment. The problem has been a lack of engagement with the available empirical data. When the Labour government was implementing the DSPD pilot, their Planning and Delivery Guide (Ministry of Justice, 2005) had a separate section on ‘Legal Context’ – which stated (p. 2):

Pilot DSPD services are operating within the ambit of current mental health and criminal justice legislation. It is however an evolving picture. The Criminal Justice Act 2003 introduced new indeterminate sentences for dangerous offenders whose eligibility for release will be dependent on the level of risk they pose in terms of sexual and/or violent re-offending … We will keep under review how the treatment and management of dangerous offenders can be helped by changes in the law.

In part, the coalition government were hostage to the sudden death knell of the IPP: the OPDPP was released (DH and MJ, 2011) only months prior to the introduction of the EDS. However, the problems with the IPP had been patently clear for some years; reform had been imminent for some time. The absence of serious discussion of the implications of its replacement, when significant money was being reinvested to buttress existing high-secure prison services, may prove to be a missed opportunity to set the foundations for treatment engagement among SPD-offenders.

Might part of the problem be generally unwillingness among the government, and the dedicated medical practitioners advising them, to problematise the proposed investment in treatment by predicating its potential success on external factors? This seems to me to be an unfortunate cost of cloaking the dangerous SPD-offender in manifest immorality that we refuse to relate to. Few among the law abiding have the introspective skills to admit that the quality of life of others could be improved by submitting to treatment aimed at effecting a new self-understanding; yet, most, if not all, of us would seriously consider such treatment if coerced. As one psychologist sensibly put it: ‘You don’t need [to have SPD] to have [that] different motivation. You could understand it’. Thus, coercive law is both a sword and shield.

In this paper, it has been argued that the upshot of the lack of coercion provided by the EDS is that public protection will be effected by extending the detention of SPD offender in hospital, by warrant of the Secretary of State for Justice under section 47 of the MHA. For medical practitioners, the result of working with offenders experiencing late transfers has often been chronic stress (MJ, 2011b). This is a further way in which the government suffers from a lack of joined up thinking on the issues in policy it (re-) produces.

More widely, the KUF appears to have achieved encouraging results in increasing knowledge of personality disorder, and building efficacy, compassion and capability among workforces (Davies, Sampson, Beesley, Smith, & Baldwin, 2014). However, it is hard to imagine those same workforces gaining job satisfaction when all they have in common with their patients is a desire to leave hospital.

## Disclosure statement

No potential conflict of interest was reported by the author.
